# Change of direction and linear speed relation to functional ability and joint mobility in Polish U19 volleyball and basketball 3 × 3 national teams

**DOI:** 10.3389/fspor.2024.1443224

**Published:** 2024-09-06

**Authors:** Zuzanna Czyznielewska, Tomasz Gabrys, Fatma Hilal Yagin, Ladislav Cepicka

**Affiliations:** ^1^ Department of Athletics, Middle Tennessee State University, Murfreesboro, TN, United States; ^2^ Department of Physical Education and Sport, Faculty of Pedagogy, University of West Bohemia, Pilsen, Czechia; ^3^ Department of Biostatistics and Medical Informatics, Faculty of Medicine, Inonu University, Malatya, Türkiye

**Keywords:** team sport, sprint, agility, cutting, physical fitness, performance

## Abstract

The purpose of this study was to determine the extent of differences in the level of change in linear speed and velocity in the modified change of direction test (COD) and to determine the relationship between speed deficits resulting from changes of direction and functional performance between groups of Polish U19 Volleyball National Team and Polish Women's Basketball 3 × 3 National Team. A total of 23 athletes: 12 volleyball players (age: 18 ± 0 years; body height: 183 ± 7 cm; body weight: 70 ± 8 kg) and 11 basketball players (age: 26 ± 4 years; body height: 180 ± 6 cm; body weight: 73 ± 10 kg) participated in the study. Athletes were tested for the following measures: Functional Movement Screen test (FMS), dynamic balance test Y-Balance, joints range of motion measurements, maximal sprint test (14 m), modified COD test (14 m) and change of direction deficit (CODD). A value of *p* < 0.05 was considered statistically significant. There was no significant correlation between sprint and CODD results in basketball team. In volleyball team there was a positive and significant correlation between COD, sprint and CODD. There was a negative and significant correlation between Y-Balance scores and sprint test results in the basketball team. Basketball team had a positive significant correlation between hip rotations and COD results. There was a negative significant correlation between shoulder movements and COD and CODD results in volleyball team.

## Introduction

In numerous team sports, the movement of the player is based on rapid changes of direction (COD) combined with frequently repeated sprints (1, 2). Changes in direction of movement are conditioned by the actions of the opponent, the tactics of the team or the current sports result. Factors affecting the timing of directional changes are the speed of the run-up, the angle of directional change, and the power generated during running (3). In addition, COD time is influenced by the orientation of the body position determined by the direction in which the lower limbs and hip are pointed during movement (4). Especially in impact sports, such as volleyball, this direction is often determined by the position of the opponent relative to the net. Spiteri et al. (4) and Suchomel et al. (3) emphasize the importance of leg muscle strength for fast COD. In addition, somatic build and age of athletes appear to influence COD performance (4, 5). The speed of moving with changes in direction and the speed of sprinting are determined by the generation of force relative to the ground and the shortest possible ground contact time during each step (3). However, unlike sprinting, in team games, directional changes occur in different planes, at different angles, transitioning from forward movements to lateral or backward movements (1, 2). Depending on the specific sport and position, some COD patterns occur more frequently than others (4, 5). Athletes with a high ability to change direction have an advantage in offensive and defensive situations (4). Successful changes of direction that are unpredictable to the opponent determine victory in team games. This is crucial, particularly in the context of team sports, because in team sports, change of direction is the foremost requirement for players (6).

The literature on the relationship between age and COD performance is very limited. In the work of Loturco et al. (45) an analysis of the speed in the COD test of soccer players aged U15, U17, U20, and Senior was performed. The authors note that the COD result is undoubtedly influenced by the level of dynamic force—power, which is related to the need to accelerate multiple times over a short distance (7). The biological development supporting this motor characteristic in women ends before the age of 18 (7). Therefore, this factor did not differentiate our subjects. Of course, there remains the issue of training experience, which in the case of the senior 3 × 3 basketball team examined was longer than in the case of the junior volleyball team. However, taking into account that senior teams include players aged 18 to 35, maintaining the division into age groups would not be possible to standardize senior teams in terms of test results. An additional argument for the possibility of comparing both groups was the fact that the players of both teams played in league matches in their sports disciplines at the same level of competition (first league). Therefore, the biological factor equally supported the process of improving running power, and the athletes represented the same sports level adequate to their disciplines (8).

Volleyball is a sport in which players must react in a dynamically changing and unpredictable environment (9). During the game, players constantly adjust their movements to temporal and spatial assumptions, and consequently develop eye-controlled motor coordination mechanisms. Reactive maneuvers in this sport require the integration of functional fitness with motor components (10). In volleyball, 83.7% of actions last less than 10 s, with men's actions being shorter than women's. The distances covered, are 85.3% less than 15 meters including 45.7% between 5 and 10 meters (11). Therefore, sprint and COD tests should be tailored to volleyball-specific requirements (12). Volleyball players mainly cover short distances, and lateral movements often occur after only a few steps of forward running.

3 × 3 basketball is a sport that features a high intensity workload in a relatively short period of time. One game lasts 15 to 20 min, and each player has his individual average time, which he spends either playing or sitting on the bench. Consider that the number of measured directional changes in 3 × 3 basketball is much higher than in the classic variety of this sport, as it consists of more lateral and backward movements. Montgomery and Maloney (46) analyzed data from elite 3 × 3 games and found that elite players made 32 accelerations, 34 decelerations and 11 directional changes during a single court entry. The facts highlighted earlier indicate that COD speed is one of the most important determinants of success in basketball (13).

The relationship between functional abilities and performance indicators remains unclear. An investigation into whether functional status scores are related to tests of speed (sprint) and changes of direction (COD) is warranted to verify these ambiguities. Evidence on whether specific scores on the Functional Movement Screening test (FMS), Y-Balance test or joints range of motion measurements have associations with athletic performance is limited (14, 15). Despite research in this area, the relationship between functional fitness and speed and change of direction profiles has not been fully elucidated (14, 15). Evidence is lacking regarding the relationship between functional testing and kinematic performance: linear speed (sprint), COD ability, change of direction deficit (CODD) in elite athletes.

A limited number of studies have examined the relationship between linear sprinting and COD deficits in groups of team sport athletes (16–18). Freitas et al. (8) showed using a comparison of players’ acceleration magnitudes that players who accelerated faster produced a higher COD deficit. Additionally, Loturco et al. (16) pointed to the implementation of special training programs that would emphasize replicating situations similar to those in real games, which also, have a positive impact on reducing the COD deficit (5, 19, 20).

The work of Hernández-Davó et al. and Gonzalo-Skok et al. highlighted the relationship of the ability to change direction with a small loss of speed to functional status.

Research from Opplert et al. on the impact of muscle mobility on the performance of athletes showed that there is strong evidence confirming the positive or neutral effect of dynamic stretching on muscle performance (21).

The study undertaken was to determine the extent of differences in the level of change in linear speed and velocity in the modified COD test between groups of Polish U19 Volleyball National Team and Polish Women's Basketball 3 × 3 National Team. The second objective was to determine the relationship between speed deficits resulting from changes of direction and functional performance in female national team level volleyball and 3 × 3 basketball athletes.

The hypotheses of the study were that it will be a correlation between the functional status as assessed by the FMS test, Y-Balance test and joints range of motion measurements and the change of direction (COD) ability, change of direction deficit (COD deficit) and linear speed (sprint) in elite 3 × 3 basketball and volleyball players (U19). And there will also be differences in the level of change in linear speed and velocity in the modified COD test between groups of Polish U19 Volleyball National Team and Polish Women's Basketball 3 × 3 National Team.

## Materials and methods

### Experimental approach to the problem

A cross-sectional design was performed in this study to estimate the relationship between chosen variables related to COD and CODD. First part of the study included functional assessment. At the second part subjects performed a maximum running test over a distance of 14 meters and a change of direction test—CODAT, in modification of the purpose of the study, also over a distance of 14 meters. The activities were to examine:

– Differences in the level of change in linear speed and speed in the modified COD test between groups of volleyball (U19) and basketball 3 × 3 players at the level of the Polish National Team,
– Relationships between speed deficits resulting from changes of direction and functional status in female national team members in volleyball (U19) and 3 × 3 basketball.

### Subjects

The study included 23 female members of Polish National Team in volleyball (U19) and basketball 3 × 3. The characteristics of the study groups are shown in Table 1. Before taking part in the study, each participant was informed of the purpose of the study and the confidentiality of the results. Participants were free to withdraw their participation in the study without any consequences. The study was approved by the institutional bioethics committee of the Jan Długosz University in Częstochowa (KE-U/2/2021) and the subjects were informed of the benefits and risks of the investigation prior to signing an institutionally approved informed consent document to participate in the study. Additionally, for every subject who was under the age of 18 years old, parental or guardian signed consent was obtained. Inclusion criteria were: consent to participate in the study, good health and active participation in a regular training regimen. Exclusion criteria were: lack of permission from the coach, injury or illness, and rehabilitation that did not allow the participant to take part in regular training. The study was conducted during a normal training week. Twenty-four hours before the study, the athletes did not perform any physical exertion, did not use ergogenic agents and did not take caffeine-containing preparations.

**Table 1:** Characteristic of the athletes.

Variable	Team		*p*-value	ES
	Basketball 3 × 3 (*n* = 11)	Volleyball U19 (*n* = 12)		
	Mean ± SD	Mean ± SD		
Age	26 ± 4	18 ± 0	**<0**.**001**	0.045
Height (cm)	180 ± 6	183 ± 7	NS	–
Weight (kg)	73 ± 10	70 ± 8	NS	–

SD, standard deviation; NS, not-significant; ES, effect size.

### Procedures

The procedures were carried out in March of 2022, during the regular season for both teams. The study was conducted in two parts. The first part of the study: assessment of functional status, based on tests: FMS, Y-Balance and joints range of motion measurements. All measurements were carried out from 8 a.m. to 12 p.m., keeping the order of subjects constant. The second part of the study: sprint (14 m) and modified COD (14 m) tests, were conducted the following day from 8:00 a.m. to 12:00 p.m., keeping the same order of subjects. Throughout all the tests, players were instructed to perform their maximum effort and were verbally encouraged by the research team. The standardized warm-up in all testing sessions consisted of dynamic warm-up progressing from general to sport-specific movement patterns to prepare the athletes for activity. General movements during the warm-up aimed to activate total body musculature in the different planes of motion (e.g., lunge and twist, reverse lunge and overhead reach, world's greatest stretch, lateral lunges, inchworms, knee hugs, ankle pulls, and side sweeps). Sport-specific activities included the carioca, high knees, skips for maximal height and distance, and drills involving accelerations, decelerations, and COD actions (such as a sprint, side shuffle right, backpedal, side shuffle left, and sprint to the end line). Following the dynamic warm-up, the team would transition into two submaximal sprints over 14 m, and two maximal sprints over the same distance.

In all experiments, exclusion criteria were as follows: (a) potential medical problems or an injury history of ankle, knee, or back pathology in the last three months prior to the study; (b) previous medical, drug or orthopaedic problems that compromised their participation or performance in this study.

In all experiments, participants were instructed to maintain their daily habits (e.g., exercise, nutrition) for the whole duration of the study. In all experiments, before signing an informed consent document, all the participants were carefully informed about the experiment procedures, the potential risk and benefits associated with participation in the study.

#### Anthropometric measurements

All anthropometric measurements were taken according to standard methods: body height (to the nearest 0.1 cm), body weight (0.1 kg). Participants wore light indoor clothing and were barefoot when measured. Measurements were taken using a METRISIS anthropometer and a TANITA MC-780 P MA scale.

#### Functional movement screen test—FMS

The FMS concept provides a simple, accessible and quantifiable way to assess the quality of movement patterns and identify any limitations or asymmetries in subjects. The FMS consists of 7 basic items, during which disorders in a given kinematic chain are highlighted (22). These tests assess joint mobility, muscle flexibility, stability, coordination and balance skills (23). Individuals who have difficulty performing the tests may develop compensatory patterns during motor activity that limit maximum physical capabilities, leading to biomechanical movement disorders with consequent injury.

The FMS assessment was performed without a warm-up, and athletes wore athletic attire and footwear. Specialized equipment was used to conduct the test: “KITM” (manufacturer: Technomex). The kit included a base: plank with dimensions of 5, 15, 150 cm, tubes with centimeter scale and rubber cord (24).

#### Y-Balance test

The Y-Balance test is performed by dividing the body into four parts (lower quarter-right side, lower quarter-left side, upper quarter-right side, upper quarter-left side) and for functional diagnosis of the spine and each limb under the influence of body weight (25, 26). The device and protocol are highly accurate and can be used to measure dynamic balance for physically active individuals (27). The Y-Balance dynamic balance test consists of two components:

– lower body,
– upper body.

The test subject performs 3 tests in each of the 3 directions, for each part (lower and upper) and for each limb (right and left), and the maximum reach in each direction is used for analysis. The composite score of the test, the sum of the maximum reach in each of the three directions of the reach divided by three times the length of the limb, and then multiplied by 100. It gives a picture of the overall performance of the test subject and refers to his body (27).

The Y-Balance dynamic assessment was performed without a warm-up, and athletes wore athletic attire and footwear. Specialized equipment was used to conduct the test: “KIT” (manufacturer: Technomex).

#### Joints range of motion measurements

The basis of this part of the diagnostic was a thorough examination of the ranges of motion in individual joints, in order to obtain basic information about the athlete's skeletal system and posture (28). The goal was to gain information that would identify limitations, dysfunctions and asymmetries in the athlete's posture and movement. These limitations can negatively affect athletic performance and exercise quality and significantly increase the risk of injury or trauma (29, 30).

The most important joints and muscles were analyzed, whose dysfunction can affect the subjects’ performance, economy of movement, and increase the risk of injury.

The diagnostics consist of 18 measurements that provide a picture of an athlete's biomechanics and allow us to effectively help improve the athlete's body:

– shoulder internal rotation: left and right arm,
– shoulder external rotation: left and right arm,
– shoulder flexion: left and right arm,
– shoulder extension: left and right arm,
– upper back rotation: left and right side,
– hip internal rotation: left and right leg,
– hip external rotation: left and right leg,
– foot dorsiflexion: left and right foot,
– foot plantarflexion left and right foot.

Joints range of motion measurements were taken without a warm-up, and athletes wore athletic attire. The Gyko inertial sensor (manufacturer: Microgate), which has been introduced in sports science (31, 32), was used for the assessment. Before the measurements began, the device was mounted in a safety band and placed around the measured test segment, depending on the movement that was to be tested. The range of motion module was used for the test. All measurements of joint range of motion angles were given in degrees.

#### Maximal sprint test

**Figure 1:**
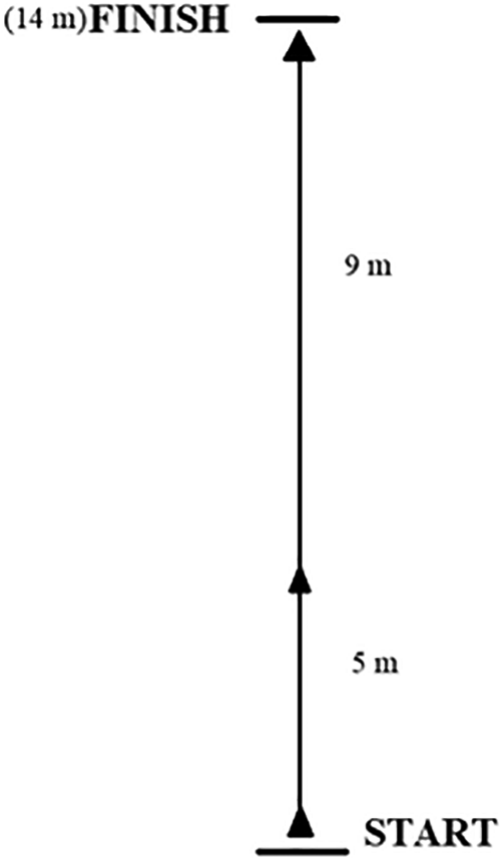
Dimensions of the maximal sprint test.

The performance of the speed effort was preceded by a standard 20 min warm-up. The subjects performed a 14-meter sprint from a standing position 3 times. Timing gates were placed at the beginning, 5th and 14th meters of the distance. Time was recorded using the SmartSpeed Pro Timing Gates system (Fusion Sport, USA). Timing results were done at the 5 m and 14 m distances. No instructions were given on the most efficient movement technique, and the subjects were instructed to run the distance as fast as possible. Linear speed analysis typically involves recording data over multiple trials. Accurate data collection requires consistency across these trials (33). So, 3 trials of each sprint were performed, after which the best result was selected for analysis (28). The dimensions of the test are shown in Figure 1.

#### Modified change of direction test—COD

**Figure 2:**
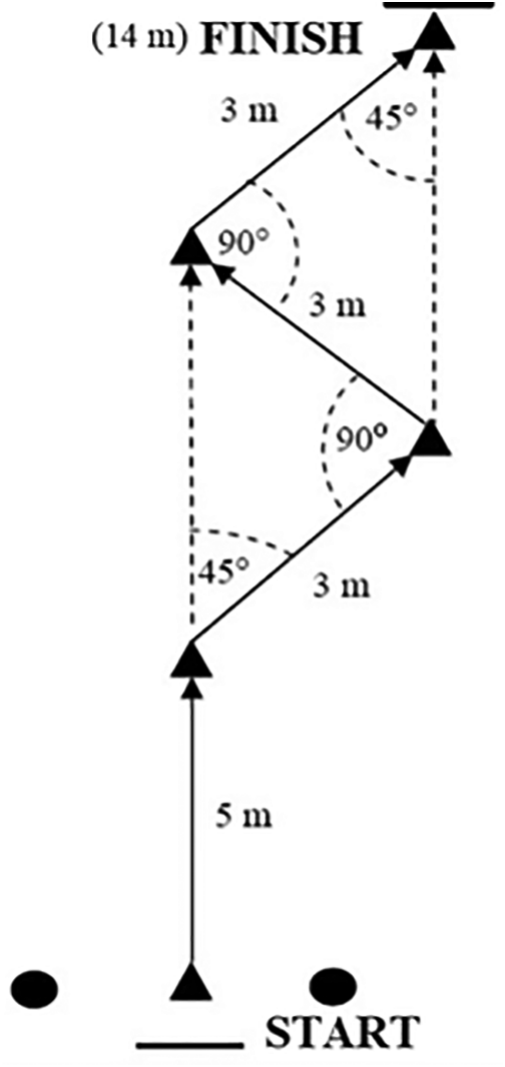
Dimensions of the modified COD test.

The change of direction (COD) test was designed based on research analyzing movement over time in team sports. It assesses the ability to change direction while sprinting forward. The study used a modification of the standard COD test. The modifications consisted of removing a 10-meter section covered in a straight line in the final part of the test. The modified change of direction test performed by the subjects included a 5-meter sprint in a straight line, followed by three 3-meter sprints performed at 45° and 90°. The dimensions and direction of movement for the COD are shown in Figure 2.

Performance of the test was preceded by a standard 20 min warm-up. Time was recorded using the SmartSpeed Pro Timing Gates system (Fusion Sport, USA). Timing results were done at 5 m and 14 m distances. No instructions were given on the most effective movement technique, and subjects were instructed to complete the test as quickly as possible (34).

Analysis of linear velocity and rate of change of direction usually involves recording data over multiple trials. Accurate data collection requires consistency across these trials (33). So, 2 sets were performed, with 5 repetitions of the test in each, where a 30 s rest was used between repetitions, and 6 min of rest were taken after the entire set.

**Figure 3:**
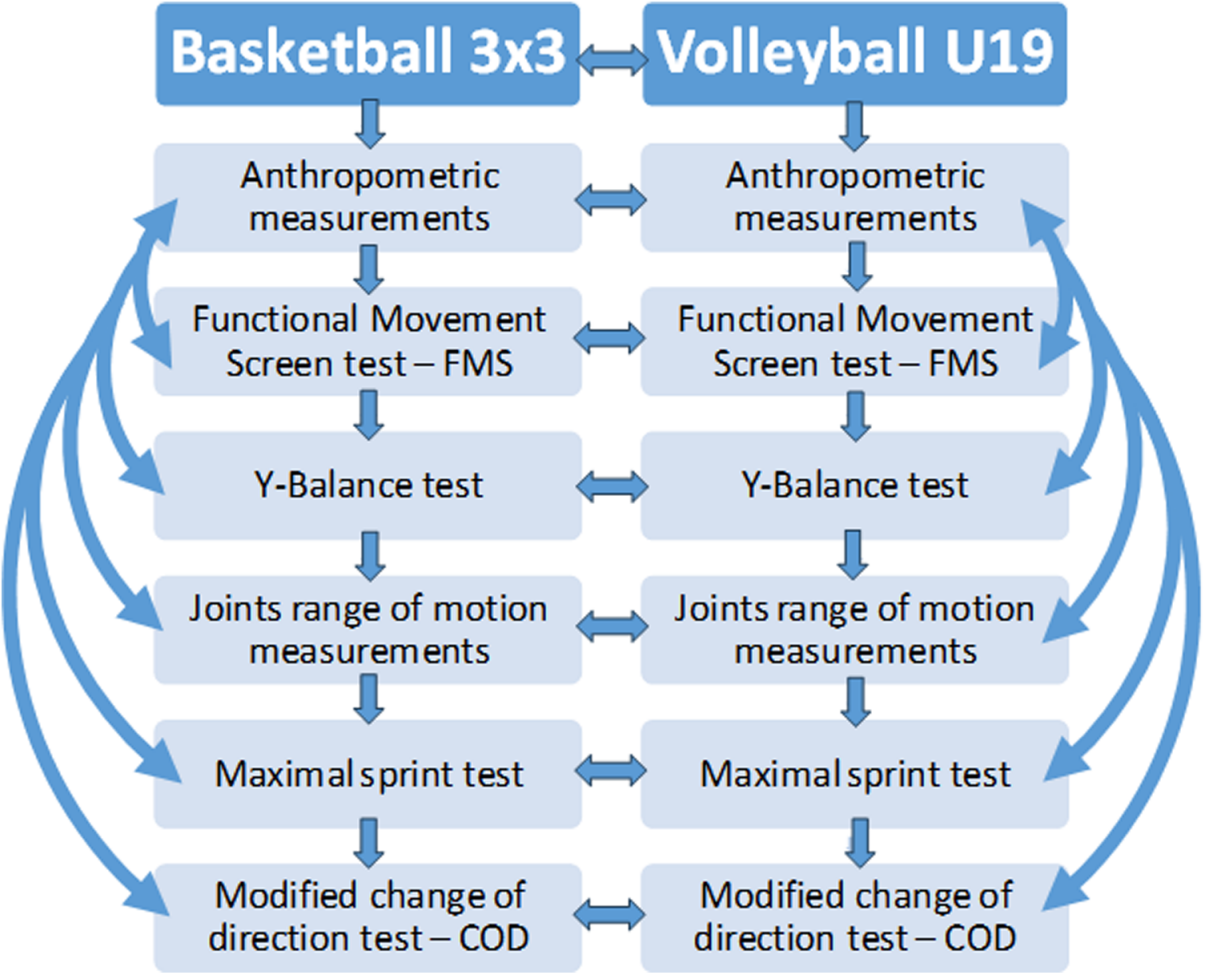
Measurement's methods and their relations between each other.

All of the measurement's methods and their relations between each other was presented in Figure 3.

### Statistical analyses

The conformity of the variables to the normal distribution was examined by visual (histogram and probability graphs) and analytical (Shapiro-Wilk Test) methods. The assumption of homogeneity of variances was examined with the Levene test. Descriptive statistics were expressed as mean ± standard deviation as quantitative data were normally distributed. Independent samples *t*-test was used to examine whether there was a significant difference between the teams in terms of FMS, Y-balance, joints range of motion measurements, sprint and COD test results. The effect size was calculated using Cohen's D. The size of the effect size was assessed by following the thresholds: Cohen considers *d* = 0.2 to be a “small” effect size, 0.5 represents a “moderate” effect size and 0.8 represents a “large” effect suggested that he did (35). Dimension. Relationships between results were made using the Pearson-r product moment correlation test. The size of the correlations was defined as: <0.1 = insignificant, 0.1–0.3 = small, 0.3–0.5 = moderate, 0.5–0.7 = large, 0.7–0.9 = very large and >0.9 = almost perfect (36). A value of *p* < 0.05 was considered statistically significant. Statistical differences were reported using the American Psychological Association (APA) 6.0 style. Analyzes were performed using the SPSS 28.0 (IBM Corp., Armonk, NY, USA) package program.

## Results

There was no significant difference between the basketball 3 × 3 and volleyball teams in terms of the results of the sprint test (*p* > 0.05) (Table 2).

**Table 2:** Results of the athletes’ maximal sprint test.

Variable	Team		*p*-value	ES
	Basketball 3 × 3	Volleyball U19		
	Mean ± SD	Mean ± SD		
5 m (s)	1.13 ± 0.08	1.11 ± 0.11	NS	–
9 m (s)	1.36 ± 0.06	1.33 ± 0.04	NS	–
14 m (s)	2.49 ± 0.13	2.44 ± 0.15	NS	–

SD, standard deviation; NS, not-significant; ES, effect size.

There was no statistically significant difference between the teams in the COD test results (*p* > 0.05) (Table 3).

**Table 3:** Results of the athletes’ modified change of direction test.

Variable	Team		*p*-value	ES
	Basketball 3 × 3	Volleyball U19		
	Mean ± SD	Mean ± SD		
5 m 1 (s)	1.24 ± 0.05	1.24 ± 0.09	NS	–
5 m 2 (s)	1.24 ± 0.07	1.25 ± 0.08	NS	–
9 m 1 (s)	3.68 ± 0.17	3.67 ± 0.24	NS	–
9 m 2 (s)	3.65 ± 0.17	3.66 ± 0.23	NS	–
14 m 1 (s)	4.93 ± 0.2	4.91 ± 0.33	NS	–
14 m 2 (s)	4.9 ± 0.18	4.91 ± 0.3	NS	–
COD deficit (s)	2.40 ± 0.14	2.44 ± 0.17	NS	–

SD, standard deviation; NS, not-significant; ES, effect size.

The results of the age, body height, body weight and FMS test of the participants are presented in Table 4. There was no statistically significant difference between the teams in FMS test results (*p* > 0.05).

**Table 4:** Results of the athletes’ functional movement screening test.

Variable	Team		*p*-value	ES
	Basketball 3 × 3	Volleyball U19		
	Mean ± SD	Mean ± SD		
Height (cm)	180 ± 6	183 ± 7	NS	–
Age	26 ± 4	18 ± 0	**<0**.**001**	0.045
Weight (kg)	73 ± 10	70 ± 8	NS	–
Deep Squat	2 ± 0	2 ± 1	NS	–
Hurdle Step-L	3 ± 0	3 ± 0	NS	–
Hurdle Step-R	3 ± 0	3 ± 0	NS	–
In-Line Lunge-L	3 ± 0	3 ± 0	NS	–
In-Line Lunge-R	3 ± 0	3 ± 0	NS	–
Shoulder Mobility-L	3 ± 0	3 ± 0	NS	–
Shoulder Mobility-R	3 ± 0	3 ± 0	NS	–
Active Straight-Leg Raise-L	3 ± 0	3 ± 0	NS	–
Active Straight-Leg Raise-R	3 ± 0	3 ± 0	NS	–
Trunk Stability—PUSH UP	3 ± 1	3 ± 0	NS	–
Rotary Stability-L	2 ± 0	2 ± 0	NS	–
Rotary Stability-R	2 ± 0	2 ± 0	NS	–
End Result	19 ± 1	19 ± 1	NS	–

R, right; L, left; SD, standard deviation; NS, not-significant; ES, effect size.

The changes of the results of the Y-Balance test according to the teams are as in Table 5. Volleyball anterior-right—lower quarter, anterior-left—lower quarter, length of right limb—upper quarter and inferolateral-left—upper quarter results were significantly higher than the basketball 3 × 3 team (*p* < 0.05).

**Table 5:** Results of the athletes’ Y-Balance test.

Variable	Team		*p*-value	ES
	Basketball 3 × 3	Volleyball U19		
	Mean ± SD	Mean ± SD		
Length of Right Limb—Lower Quarter (cm)	96.82 ± 4.58	99 ± 4.97	NS	–
Anterior—Right—Lower Quarter	62.55 ± 6.11	71.25 ± 9.28	**0**.**016**	0.015
Anterior—Left—Lower Quarter	64.45 ± 7.87	72.42 ± 7.9	**0**.**025**	0.014
Posteromedial—Right—Lower Quarter	125.45 ± 12.46	118.58 ± 10.09	NS	–
Posteromedial—Left—Lower Quarter	125.09 ± 13.34	118.58 ± 8.43	NS	–
Posterolateral—Right—Lower Quarter	123.27 ± 10.78	118.08 ± 12.34	NS	–
Posterolateral—Left—Lower Quarter	123.27 ± 15.34	117.17 ± 8.52	NS	–
Length of Right Limb—Upper Quarter (cm)	88.82 ± 2.68	91.67 ± 2.53	**0**.**016**	0.011
Medial—Right—Upper Quarter	89.55 ± 5.85	88.92 ± 5.48	NS	–
Medial—Left—Upper Quarter	90.36 ± 7.92	89 ± 4.79	NS	–
Inferolateral—Right—Upper Quarter	82.82 ± 5.31	87.75 ± 7.63	NS	–
Inferolateral—Left—Upper Quarter	81.55 ± 6.42	88.25 ± 8.3	**0**.**043**	0.012
Superolateral—Right—Upper Quarter	76.18 ± 7.37	72.17 ± 7.66	NS	–
Superolateral—Left—Upper Quarter	74.73 ± 8.93	72.75 ± 11.06	NS	–
Result—Right—Lower Quarter	107.82 ± 6.85	103.83 ± 9.71	NS	–
Result—Left—Lower Quarter	107.55 ± 7.92	104.08 ± 8.16	NS	–
Result—Right—Upper Quarter	93.18 ± 5.56	90.42 ± 3.8	NS	–
Result—Left—Upper Quarter	92.64 ± 7.16	91.08 ± 5.73	NS	–

SD, standard deviation; NS, not-significant; ES, effect size; Bold values denote statistical significance at the *p* < 0.05 level.

The changes in the joints range of motion measurements in the teams were given in Table 6. The effect of the teams was important in all of the upper body measurements (*p* < 0.05). Results showed that the results of the joints range of motion measurements in volleyball team were higher than the basketball 3 × 3 team.

**Table 6:** Results of the athletes’ joints range of motion measurements.

Variable	Team		*p*-value	ES
	Basketball 3 × 3	Volleyball U19		
	Mean ± SD	Mean ± SD		
Shoulder Internal Rotation—Left Arm (°)	54.09 ± 1.76	64.5 ± 4.19	**<0**.**001**	0.017
Shoulder Internal Rotation—Right Arm (°)	55.45 ± 2.11	65.25 ± 4.54	**<0**.**001**	0.016
Shoulder External Rotation—Left Arm (°)	111.55 ± 2.62	123 ± 8.4	**<0**.**001**	0.008
Shoulder External Rotation—Right Arm (°)	111 ± 1.79	123 ± 7.82	**<0**.**001**	0.008
Shoulder Flexion—Left Arm (°)	170.36 ± 2.66	188.08 ± 5.48	**<0**.**001**	0.005
Shoulder Flexion—Right Arm (°)	168.64 ± 2.5	187.08 ± 4.32	**<0**.**001**	0.006
Shoulder Extension—Left Arm (°)	74.91 ± 1.92	88.5 ± 4.01	**<0**.**001**	0.012
Shoulder Extension—Right Arm (°)	74.18 ± 1.66	84.58 ± 4.27	**<0**.**001**	0.012
Upper Back Rotation—Left Side (°)	38.09 ± 2.47	49 ± 4.55	**<0**.**001**	0.023
Upper Back Rotation—Right Side (°)	37.36 ± 1.36	48.83 ± 3.88	**<0**.**001**	0.023
Hip Internal Rotation—Left Leg (°)	55.36 ± 0.92	53.33 ± 3.47	NS	–
Hip Internal Rotation—Right Leg (°)	53.91 ± 2.02	52.83 ± 2.76	NS	–
Hip External Rotation—Left Leg (°)	50.64 ± 4.03	48.75 ± 2.96	NS	–
Hip External Rotation—Right Leg (°)	49.36 ± 2.01	48.58 ± 4.34	NS	–
Foot Dorsiflexion- Left Foot (°)	55 ± 3.63	53.83 ± 3.76	NS	–
Foot Dorsiflexion- Right Foot (°)	52.91 ± 1.87	51.83 ± 2.69	NS	–
Foot Plantarflexion- Left Foot (°)	47.82 ± 3.92	49 ± 3.02	NS	–
Foot Plantarflexion- Right Foot (°)	47.27 ± 3.52	47.33 ± 3.28	NS	–

SD, standard deviation; NS, not-significant; ES, effect size; Bold values denote statistical significance at the *p* < 0.05 level.

The basketball 3 × 3 team showed a negative and significant correlation between body height and the end result of the FMS test, but there was a positive correlation between the body height and the COD deficit result. COD 14 m results with sprint 14 m, and COD deficit results were found to be a positive correlation between the results. However, there was no significant correlation between sprint 14 m and COD deficit results in the basketball 3 × 3 team. In volleyball team there was a positive and significant correlation between COD 14 m, sprint 14 m and COD deficit (Table 7).

**Table 7:** Results of correlation analysis between FMS, COD and sprint tests in teams.

Team	Variable	Statistics	Height (cm)	Age	WeighT (kg)	End result	COD 1–14 m 1 (s)	COD 2–14 m 2 (s)	Sprint-14 m (s)	COD Deficit (s)
Basketball 3 × 3	Height (cm)	*r*	1	−0.012	0.753	−0.776	0.542	0.598	0.130	0.667
		*p*-value		0.972	**0**.**007**	**0**.**005**	0.085	0.052	0.704	**0**.**025**
	Age	*r*		1	−0.419	−0.171	0.252	0.072	0.516	−0.328
		*p-*value			0.200	0.616	0.454	0.834	0.105	0.325
	Weight (kg)	*r*			1	−0.644	0.060	0.185	−0.253	0.444
		*p*-value				**0**.**032**	0.861	0.587	0.453	0.172
	End Result	*r*				1	−0.356	−0.316	0.058	−0.498
		*p-*value					0.283	0.344	0.865	0.119
	COD 1–14 m 1 (s)	*r*					1	0.963	0.696	0.648
		*p-*value						**<0**.**001**	**0**.**017**	**0**.**031**
	COD 2–14 m 2 (s)	*r*						1	0.655	0.717
		*p*-value							**0**.**029**	**0**.**013**
	Sprint-14 m (s)	*r*							1	−0.053
		*p-*value								0.877
	COD Deficit (s)	*r*								1
		*p-*value								
Volleyball U19	Height (cm)	*r*	1	−0.426	0.797	0.113	0.486	0.473	0.455	0.425
		*p*-value		0.167	**0**.**002**	0.726	0.109	0.120	0.138	0.169
	Age	*r*		1	−0.272	−0.290	−0.175	−0.111	0.015	−0.222
		*p-*value			0.393	0.360	0.586	0.732	0.964	0.488
	Weight (kg)	*r*			1	0.017	0.015	−0.019	−0.057	0.001
		*p*-value				0.957	0.964	0.953	0.860	0.997
	End Result	*r*				1	0.106	0.046	−0.022	0.110
		*p-*value					0.744	0.887	0.945	0.733
	COD 1–14 m 1 (s)	*r*					1	0.972	0.879	0.968
		*p*-value						**<0**.**001**	**<0**.**001**	**<0**.**001**
	COD 2–14 m 2 (s)	*r*						1	0.936	0.931
		*p*-value							**<0**.**001**	**<0**.**001**
	Sprint-14 m (s)	*r*							1	0.759
		*p-*value								**0**.**004**
	COD Deficit (s)	*r*								1
		*p*-value								

*r* = Pearson correlation coefficient; Bold values denote statistical significance at the *p* < 0.05 level.

In the basketball 3 × 3 team there was a positive significant correlation between length of right limb—lower quarter and length of right limb—upper quarter. There was a negative correlation between length of right limb—lower quarter results and result—right—upper quarter results between the results of the basketball 3 × 3. There was a negative and significant correlation between result—right—upper quarter and result—left—upper quarter results and sprint 14 m results. In the volleyball team, there was a positive correlation between length of right limb—lower quarter and length of right limb—upper quarter, while the length of right limb—lower quarter and result—left—lower quarter had a significant correlation between the high level. In addition, there was a negative significant correlation between result—right—lower quarter and result—left—lower quarter results and COD 14 m 1, sprint 14 m and COD deficit results (Table 8).

**Table 8:** Results of correlation analysis between Y balance, sprint and COD tests in teams.

Team	Variable	Statistics	Length of right limb—Lower Quarter	Length of right limb—Upper Quarter	Result—Right—Lower Quarter	Result—Left—Lower Quarter	Result—Right—Upper Quarter	Result—Left—Upper Quarter	COD 1–14 m 1 (s)	COD 2–14 m 2 (s)	Sprint-14 m (s)	COD Deficit (s)
Basketball 3 × 3	Length of Right Limb—Lower Quarter (cm)	*r*	1	0.756	−0.125	0.174	−0.623	−0.579	0.580	0.635	0.392	0.471
		*p*-value		**0**.**007**	0.713	0.609	**0**.**041**	0.062	0.061	**0**.**036**	0.233	0.144
	Length of Right Limb—Upper Quarter (cm)	*r*		1	0.123	0.402	−0.293	−0.228	0.296	0.388	0.373	0.155
		*p-*value			0.718	0.221	0.382	0.500	0.376	0.238	0.259	0.648
	Result—Right—Lower Quarter	*r*			1	0.800	0.247	0.496	0.219	0.214	0.068	0.250
		*p-*value				**0**.**003**	0.463	0.121	0.517	0.527	0.842	0.459
	Result—Left—Lower Quarter	*r*				1	0.034	0.281	0.228	0.263	0.150	0.219
		*p*-value					0.921	0.403	0.501	0.435	0.660	0.518
	Result—Right—Upper Quarter	*r*					1	0.941	−0.489	−0.433	−0.655	0.015
		*p-*value						**<0**.**001**	0.127	0.183	**0**.**029**	0.965
	Result—Left—Upper Quarter	*r*						1	−0.460	−0.424	−0.658	0.047
		*p*-value							0.155	0.193	**0**.**028**	0.891
	COD 1–14 m 1 (s)	*r*							1	0.963	0.696	0.648
		*p*-value								**<0**.**001**	**0**.**017**	**0**.**031**
	COD 2–14 m 2 (s)	*r*								1	0.655	0.717
		*p-*value									**0**.**029**	**0**.**013**
	Sprint-14 m (s)	*r*									1	−0.053
		*p*-value										0.877
	COD Deficit (s)	*r*										1
		*p*-value										
Volleyball U19	Length of Right Limb—Lower Quarter (cm)	*r*	1	0.577	−0.550	−0.611	−0.053	−0.057	0.562	0.569	0.525	0.540
		*p*-value		**0**.**049**	0.064	**0**.**035**	0.870	0.859	0.057	0.054	0.080	0.070
	Length of Right Limb—Upper Quarter (cm)	*r*		1	−0.150	−0.157	−0.031	−0.079	−0.108	−0.106	0.010	−0.202
		*p*-value			0.641	0.627	0.923	0.806	0.739	0.743	0.976	0.529
	Result—Right—Lower Quarter	*r*			1	0.914	0.359	0.154	−0.653	−0.753	−0.670	−0.639
		*p*-value				**<0**.**001**	0.252	0.633	**0**.**021**	**0**.**005**	**0**.**017**	**0**.**025**
	Result—Left—Lower Quarter	*r*				1	0.450	0.196	−0.664	−0.735	−0.590	−0.705
		*p-*value					0.142	0.541	**0**.**019**	**0**.**006**	**0**.**043**	**0**.**010**
	Result—Right—Upper Quarter	*r*					1	0.838	0.003	−0.161	−0.168	−0.049
		*p*-value						**0**.**001**	0.992	0.617	0.603	0.879
	Result—Left—Upper Quarter	*r*						1	0.157	−0.023	−0.084	0.127
		*p*-value							0.627	0.943	0.795	0.693
	COD 1–14 m 1 (s)	*r*							1	0.972	0.879	0.968
		*p*-value								**<0**.**001**	**<0**.**001**	**<0**.**001**
	COD 2–14 m 2 (s)	*r*								1	0.936	0.931
		*p*-value									**<0**.**001**	**<0**.**001**
	Sprint-14 m (s)	*r*									1	0.759
		*p-*value										**0**.**004**
	COD Deficit (s)	*r*										1
		*p*-value										

*r* = Pearson correlation coefficient; Bold values denote statistical significance at the *p* < 0.05 level.

Basketball 3 × 3 team had a positive significant correlation between hip internal rotation—right leg and COD 14 m results. Therefore, as the results of hip internal rotation—right leg increased, COD 14 m results increased. The results of the upper back rotation—left side and sprint 14 m test results were found to have a negative significant correlation. Therefore, when the results of the upper back rotation left side increased, sprint 14 m results decreased. However, there was no significant correlation between joints range of motion measurements: rotations results and sprint and COD tests in volleyball team. The results of the upper back rotation—left side and sprint 14 m test results were found to have a negative significant correlation (Table 9).

**Table 9:** Results of correlation analysis between joints range of motion measurements: rotations, sprint and COD test in teams.

Team	Variable	Statistics	Upper back rotation—left side (°)	Upper back rotation—right side (°)	Hip internal rotation—left leg (°)	Hip internal rotation—right leg (°)	Hip external rotation—left leg (°)	Hip external rotation—right leg (°)	COD 1–14 m 1 (s)	COD 2–14 m 2 (s)	Sprint-14 m (s)	COD deficit (s)
Basketball 3 × 3	Upper back rotation -left side (°)	*r*	1	0.584	−0.630	−0.259	0.175	0.234	−0.328	−0.244	−0.659	0.248
		*p*-value		0.059	**0**.**038**	0.443	0.608	0.488	0.324	0.471	**0**.**027**	0.462
	Upper back rotation -right side (°)	*r*		1	−0.116	0.267	0.118	0.312	0.190	0.231	−0.011	0.308
		*p*-value			0.735	0.427	0.731	0.351	0.576	0.495	0.975	0.357
	Hip internal rotation—left leg (°)	*r*			1	0.661	−0.256	−0.239	0.553	0.552	0.413	0.389
		*p*-value				**0**.**027**	0.447	0.478	0.078	0.078	0.207	0.237
	Hip internal rotation -right leg (°)	*r*				1	0.314	0.181	0.584	0.602	0.551	0.295
		*p*-value					0.346	0.595	0.059	**0**.**050**	0.079	0.379
	Hip external rotation—left leg (°)	*r*					1	0.449	−0.263	−0.173	0.086	−0.344
		*p-*value						0.166	0.434	0.611	0.801	0.300
	Hip external rotation -right leg (°)	*r*						1	0.338	0.355	0.199	0.273
		*p*-value							0.310	0.284	0.557	0.416
	COD 1–14 m 1 (s)	*r*							1	0.963	0.696	0.648
		*p*-value								**<0**.**001**	**0**.**017**	**0**.**031**
	COD 2–14 m 2 (s)	*r*								1	0.655	0.717
		*p*-value									**0**.**029**	**0**.**013**
	Sprint-14 m (s)	*r*									1	−0.053
		*p*-value										0.877
	COD deficit (s)	*r*										1
		*p*-value										
Volleyball U19	Upper back rotation -left side (°)	*r*	1	0.638	0.046	0.253	0.506	0.087	−0.519	−0.505	−0.571	−0.457
		*p*-value		**0**.**026**	0.887	0.427	0.093	0.787	0.084	0.094	0.052	0.135
	Upper back rotation -right side (°)	r		1	0.247	0.558	0.115	0.114	−0.373	−0.430	−0.415	−0.366
		*p*-value			0.438	0.060	0.722	0.724	0.232	0.163	0.180	0.241
	Hip internal rotation—left leg (°)	*r*			1	0.614	−0.062	0.239	−0.473	−0.380	−0.292	−0.447
		*p*-value				**0**.**034**	0.848	0.454	0.121	0.223	0.357	0.145
	Hip internal rotation -right leg (°)	*r*				1	−0.117	0.700	−0.422	−0.444	−0.456	−0.353
		*p-*value					0.717	**0**.**011**	0.172	0.148	0.137	0.260
	Hip external rotation—left leg (°)	*r*					1	0.197	−0.309	−0.217	−0.065	−0.409
		*p*-value						0.540	0.328	0.497	0.840	0.187
	Hip external rotation -right leg (°)	*r*						1	−0.370	−0.371	−0.310	−0.376
		*p*-value							0.237	0.236	0.326	0.229
	COD 1–14 m 1 (s)	*r*							1	0.972	0.879	0.968
		*p-*value								**<0**.**001**	**<0**.**001**	**<0**.**001**
	COD 2–14 m 2 (s)	*r*								1	0.936	0.931
		*p*-value									**<0**.**001**	**<0**.**001**
	Sprint-14 m (s)	*r*									1	0.759
		*p*-value										**0**.**004**
	COD deficit (s)	*r*										1
		*p*-value										

*r* = Pearson correlation coefficient; Bold values denote statistical significance at the *p* < 0.05 level.

The basketball 3 × 3 team had a negative significant correlation between shoulder extension and COD deficit results, and therefore the results of the COD deficit decreased when the results of the shoulder extension—left arm increased. There was a negative significant correlation between shoulder extension—left arm and COD 14 m and COD deficit results, but there was no significant correlation between the results of shoulder extension—left arm and sprint 14 m. In the volleyball team there was very large significant positive correlation between the sprint 14 m test and COD deficit results, but there was no significant correlation between the results of shoulder movements, COD and sprint 14 m tests (Table 10).

**Table 10:** Results of correlation analysis between joints range of motion measurements: shoulder movements, sprint and COD tests in teams.

Team	Variable	Statistics	Shoulder flexion—left arm (°)	Shoulder flexion—right arm (°)	Shoulder extension—left arm (°)	Shoulder extension—right arm (°)	COD 1–14 m 1 (s)	COD 2–14 m 2 (s)	Sprint-14 m (s)	COD deficit (s)
Basketball 3 × 3	c—left arm (°)	*r*	1	−0.370	−0.718	0.527	0.314	0.344	−0.013	0.473
		*p*-value		0.263	**0**.**013**	0.096	0.346	0.300	0.969	0.141
	Shoulder flexion—right arm (°)	*r*		1	−0.008	−0.295	−0.150	−0.322	−0.112	−0.262
		*p*-value			0.982	0.378	0.659	0.334	0.744	0.436
	Shoulder extension—left arm (°)	*r*			1	−0.495	−0.582	−0.567	−0.150	−0.656
		*p*-value				0.121	0.061	0.069	0.660	**0**.**029**
	Shoulder extension—right arm (°)	*r*				1	0.409	0.387	0.222	0.325
		*p*-value					0.211	0.239	0.511	0.330
	COD 1–14 m 1 (s)	*r*					1	0.963	0.696	0.648
		*p*-value						**<0**.**001**	**0**.**017**	**0**.**031**
	COD 2–14 m 2 (s)	*r*						1	0.655	0.717
		*p*-value							**0**.**029**	**0**.**013**
	Sprint-14 m (s)	*r*							1	−0.053
		*p*-value								0.877
	COD deficit (s)	*r*								1
		*p*-value								
Volleyball U19	Shoulder flexion—left arm (°)	*r*	1	0.622	−0.048	0.316	0.106	0.067	0.250	−0.015
		*p*-value		**0**.**031**	0.883	0.317	0.743	0.836	0.433	0.963
	Shoulder flexion—right arm (°)	*r*		1	0.181	0.337	−0.297	−0.291	−0.102	−0.383
		*p*-value			0.573	0.284	0.349	0.358	0.751	0.219
	Shoulder extension—left arm (°)	*r*			1	0.740	−0.702	−0.644	−0.546	−0.671
		*p*-value				**0**.**006**	**0**.**011**	**0**.**024**	0.066	**0**.**017**
	Shoulder extension—right arm (°)	*r*				1	−0.458	−0.498	−0.359	−0.505
		*p*-value					0.134	0.100	0.252	0.094
	COD 1–14 m 1 (s)	*r*					1	0.972	0.879	0.968
		*p*-value						**<0**.**001**	**<0**.**001**	**<0**.**001**
	COD 2–14 m 2 (s)	*r*						1	0.936	0.931
		*p*-value							**<0**.**001**	**<0**.**001**
	Sprint-14 m (s)	*r*							1	0.759
		*p*-value								**0**.**004**
	COD deficit (s)	*r*								1
		*p*-value								

*r* = Pearson correlation coefficient; Bold values denote statistical significance at the *p* < 0.05 level.

## Discussion

In team sports, players with a high ability to change direction gain an advantage in field situations (4, 37). It can determine victory in team games, so sprints and directional change tasks are a very important factor in training and physical preparation diagnostics (4, 5). Improving the efficiency of an athlete's movement on the field can be conditioned to a large extent by the level of functional status and the level of physical preparation in terms of generate power (3–5).

According to the real-life scenario of 3 × 3 basketball and volleyball, the majority of directional change maneuvers take place at angles between 0 and 90° (38, 39). This condition is met by the COD test, which was used in a study of female athletes in these two sports.

A study by Sayers (47) found that COD should be measured at shorter distances to avoid the influence of linear velocity. Therefore, the study modified the COD test by reducing it from 24 meters to a distance of 14 meters. The results obtained were related to the sprint test over a distance of 14 meters. The COD values of the deficit, which is the difference between the time results from the COD 14 m test and the time results from the sprint 14 m test, were obtained, which corresponds to the specifics of both sports.

There was no significant difference in the timing results between the 3 × 3 basketball and volleyball teams in the sprint, COD and COD deficit tests.

In 3 × 3 basketball and volleyball teams, a positive and statistically significant correlation was found between COD, sprint and deficit COD scores. These results apply to all distances and are consistent with previous studies (37, 38).

A limited number of studies have examined the relationship between linear sprinting and COD deficits in groups of team sport athletes (16–18). Our results are consistent with the findings of Papli et al. (18), where the authors showed that 20 m linear sprint time was not significantly correlated with COD deficit in two different tests. This observation is consistent with previous studies in team sports, in which participants were rugby (40, 41), soccer (8, 16) and handball players (42). More specifically, Loturco et al. (16) on a sample of 25 male soccer players, after dividing them into groups of faster and slower players, found that the faster players had higher COD deficit values. Freitas et al. (8) showed using a comparison of players’ acceleration magnitudes that players who accelerated faster produced a higher COD deficit.

Thus, we can conclude that the transition from linear speed to COD is a common problem in team sports (2). To this end, Loturco et al. (16) pointed to the implementation of special training programs that would emphasize replicating situations similar to those in real games, which also, have a positive impact on reducing the COD deficit, stressing that the athlete must exert significant forces on the ground, both horizontally and vertically, in the shortest possible time in order to continuously and efficiently perform accelerations and decelerations (5, 19, 20).

The work of Hernández-Davó et al. and Gonzalo-Skok et al. highlighted the relationship of the ability to change direction with a small loss of speed to functional status. Female athletes of the Polish National Team in volleyball (U19) and basketball 3 × 3 had high FMS scores. There was no significant difference between the teams in both the final scores and the final scores for the right and left sides.

Comparing the Y-Balance tests, we note that the volleyball team's measurements on the lower quarter-right side, lower quarter-left side, upper quarter-right side, upper quarter-left side were significantly higher than those of the 3 × 3 basketball team. Grassi et al. indicated that the Y-Balance test requires lower limb muscle strength, coordination and agility (43). These are qualities that also determine the ability to change direction quickly. Joints range of motion measurements showed that all upper-body outcomes in the volleyball team were higher than the 3 × 3 basketball team. Sport-specific adaptations in joints range of motion may occur in athletes, such as volleyball players, due to the narrow specialization of the sport practiced and the very high frequency of performing specific ranges of motion (44). Therefore, our study confirms these relationships.

However, in the work by Opplert et al. on the impact of muscle mobility on the performance of athletes, they showed that there is strong evidence confirming the positive or neutral effect of dynamic stretching on muscle performance (21).

Therefore, compared to other works, the findings in our study regarding the relation between functional ability and joint mobility and sprinting, COD and COD deficit seem to be very interesting.

From the practical application's standpoint of our analysis, each athlete's functional and movement deficits should be taken into account in order to program an appropriately individualized process to improve movement and change of direction performance.

Surprisingly, no range of differences were recorded in the level of change in linear speed and modified COD test results between the groups of volleyball and 3 × 3 basketball players.

However, linear sprint tests and change of direction (COD) tests with specific distance assessment (5, 9, 14 m) seem to be reliable tools recommended in the training process for both 3 × 3 basketball and volleyball.

We would also like to show areas that our research does not explain, and our results cannot be taken into account when making decisions regarding other than a specific sports level in the two sports we describe. The volleyball and basketball 3 × 3 players presented in our study, represent an elite level, so our analysis cannot be transposed to younger teams or teams at a basic level of their sport.

## Conclusions

In conclusion, there was a correlation between the functional status as assessed by the FMS test, Y-Balance test and joints range of motion measurements and the change of direction (COD) ability, change of direction deficit (COD deficit) and linear speed (sprint) in elite 3 × 3 basketball and volleyball players (U19).

Our analysis can provide future research directions in correlation with the relevant results of the study about the complexity of COD and COD deficit performance. Which will allow practitioners to make more specific decisions about its formation during the training process. Because traditional training processes may not be applicable to COD ability development. Instead of linear sprinting drills, more functional ability-based protocols, oriented to the specifics of basketball and volleyball and targeted to specific tasks in a given sport, should be used.

## Data Availability

The original contributions presented in the study are included in the article/Supplementary Material, further inquiries can be directed to the corresponding author.
